# Unexpected tumor response to palliative pelvic radiotherapy in mismatch repair-deficient advanced prostate cancer: a case report

**DOI:** 10.1186/s13256-020-02578-4

**Published:** 2020-12-08

**Authors:** Giovanni Aluisio, Ercole Mazzeo, Frank Lohr, Federica Fiocchi, Stefania Bettelli, Cinzia Baldessari, Maurizio Paterlini, Alessio Bruni

**Affiliations:** 1 Radiotherapy Unit, Oncology and Hematology Department, University Hospital of Modena, Modena, Italy; 2 Department of Radiology, University Hospital of Modena, Modena, Italy; 3 Department of Diagnostic Medicine and Public Health, Section of Pathology, University Hospital of Modena, Modena, Italy; 4 Oncology Unit, Department of Oncology and Hematology, University Hospital of Modena, Modena, Italy; 5 Department of Urology, University Hospital of Modena, Modena, Italy

**Keywords:** Microsatellite instability, Radiosensitivity, Prostate cancer

## Abstract

**Background:**

Mismatch-repair-deficiency resulting in microsatellite instability (MSI) may confer increased radiosensitivity in locally advanced/metastatic tumors and thus radiotherapy (RT) potentially might have a changing role in treating this subset of patients, alone or in combination with checkpoint inhibitors.

**Case presentation:**

We report a 76 year-old Italian male patient presenting with locally advanced undifferentiated prostate cancer (LAPC), infiltrating bladder and rectum. Molecular analysis revealed high-MSI with an altered expression of MSH2 and MSH6 at immunohistochemistry. Two months after 6 chemotherapy cycles with Docetaxel associated to an LHRH analogue, a computed tomography scan showed stable disease. After palliative RT (30 Gy/10 fractions) directed to the tumor mass with a 3D-conformal setup, a follow-up computed tomography scan at 8 weeks revealed an impressive response that remained stable at computed tomography after 9 months, with sustained biochemical response. To our knowledge, this is the first case of such a sustained response to low dose RT alone in high-MSI LAPC.

**Conclusions:**

Routine evaluation of MSI in patients with locally problematic advanced tumors might change treatment strategy and treatment aim in this setting, from a purely palliative approach to a quasi-curative paradigm.

## Introduction

Microsatellite instability (MSI) is characterized by mutations in repetitive DNA sequence tracts, as a consequence of an insufficient DNA mismatch repair system. Deficient DNA mismatch repair (dMMR) results from bi-allelic mutational inactivation or epigenetic silencing of any of the genes in the MMR pathway. Consequently, MSI status is used as a biomarker indicative of dMMR.

MSI has been most closely studied in colorectal cancers (CRC), where it is present in up to 15–20% of cases [1]. However many other type of cancer showed MSI, like ovarian, endometrial and gastric cancer [2].

Several studies have reported MMR protein loss and MSI also in prostate cancer (PC), especially in advanced stages (APC). The prevalence of MSI-H/dMMR in PC, however, is still unclear, with frequencies ranging from 1.2% to 12.0% in previous reports [3].

MSI appears to be influencing cancer radiosensitivity, first reported in particular for MSI CRC [4], but still not clearly observed across other histologies. There is accumulating evidence to suggest that DNA MMR proteins may influence and/or are directly involved in the DNA damage response (DDR) following radiation induced double strand breaks (DSBs). Therefore, their deficiency that characterizes MSI CRC cancers may also be linked to radiosensitivity. Because of the role of DNA MMR, MSI could be involved in multiple steps of the DDR caused by radiation, for example the detection of the DSBs, their repair and the inhibition of consecutive apoptosis [5]. Several studies using colony-forming assays demonstrated a correlation between MSI and cancer radiosensitivity, expecially for CRC [6], and also between MSI and radiation exposure and normal tissue sensitivity (patients who developed severe acute reactions) [7]. There is, however, no clear evidence in the literature regarding the influence of MSI on radiosensitivity in MSI PC.

Here we present a case of MSI APC, in whom an extraordinary radiological response after palliative radiotherapy (RT) was observed, with the tumor still being in remission 34 months after treatment.

## Case report

In April 2017 a 76-year-old Italian male was referred to the Urology Department of the University Hospital of Modena for acute prostatitis and rectal bleeding. The digital rectal examination showed a rectal mass and the colonoscopy confirmed a massive rectal mucosal infiltration by a neoplastic lesion. Pathology of rectal biopsy showed a completely undifferentiated PC (Androgen Receptor+, Prostatic Specific Antigen+) with no possibility to calculate Gleason Score.

**Fig 1:**
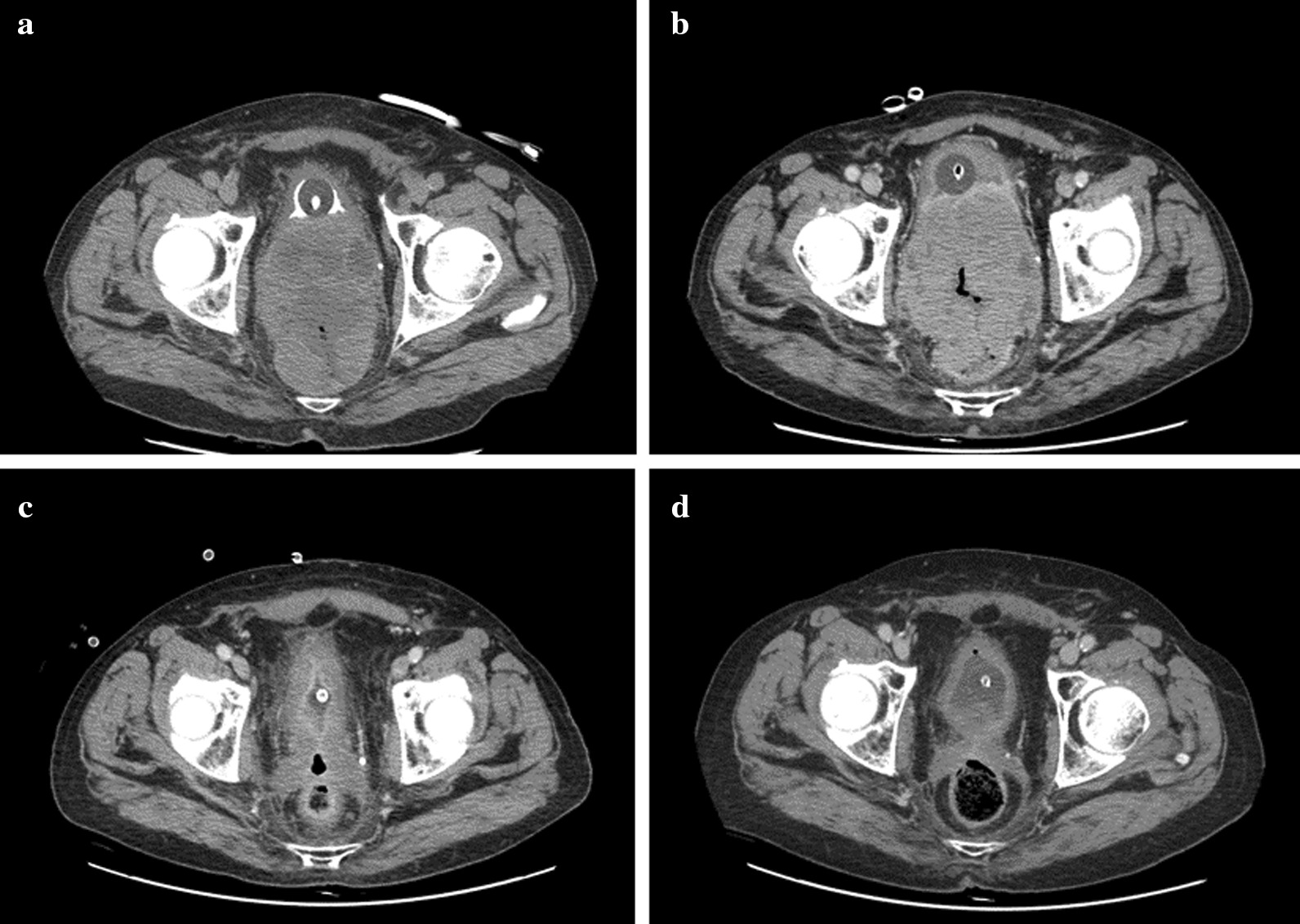
Radiographic CT response to treatment of local disease. **a** CT images of advanced disease at staging. **b** CT images after chemotherapy and LH RH analogue. **c** CT images of radiation response after two months. **d** CT images 9 months after radiation therapy

In April 2017 Magnetic Resonance Imaging and Computed Tomography (CT) scan revealed a 15 × 10 × 6 cm tumor mass which macroscopically infiltrated both bladder and rectum (Fig. 1a). Staging was completed with a bone scan that was negative for distant metastases.

At time of diagnosis Prostatic Specific Antigen value was 21.45 μg/L. As the invasion of periprostatic tissue was massive and the overall disease volume was therefore extensive, upfront radiotherapy with radical intent was considered unfeasible. The results of recent studies were then discussed with the patient and the option of an individual treatment concept was offered. From May 2017, a Luteinizing Hormone-Releasing Hormone (LH-RH) analogue and chemotherapy with Docetaxel 75 mg/m^2^ q/21d was administered as first line therapy.

In June 2017 bilateral nephrostomy became necessary, due to bladder infiltration, and a urinary catheterization was performed.

In August 2017, chemotherapy was withheld after 6 cycles, continuing with LH-RH analogue only, as suggested by the relevant international guidelines.

**Fig 2:**
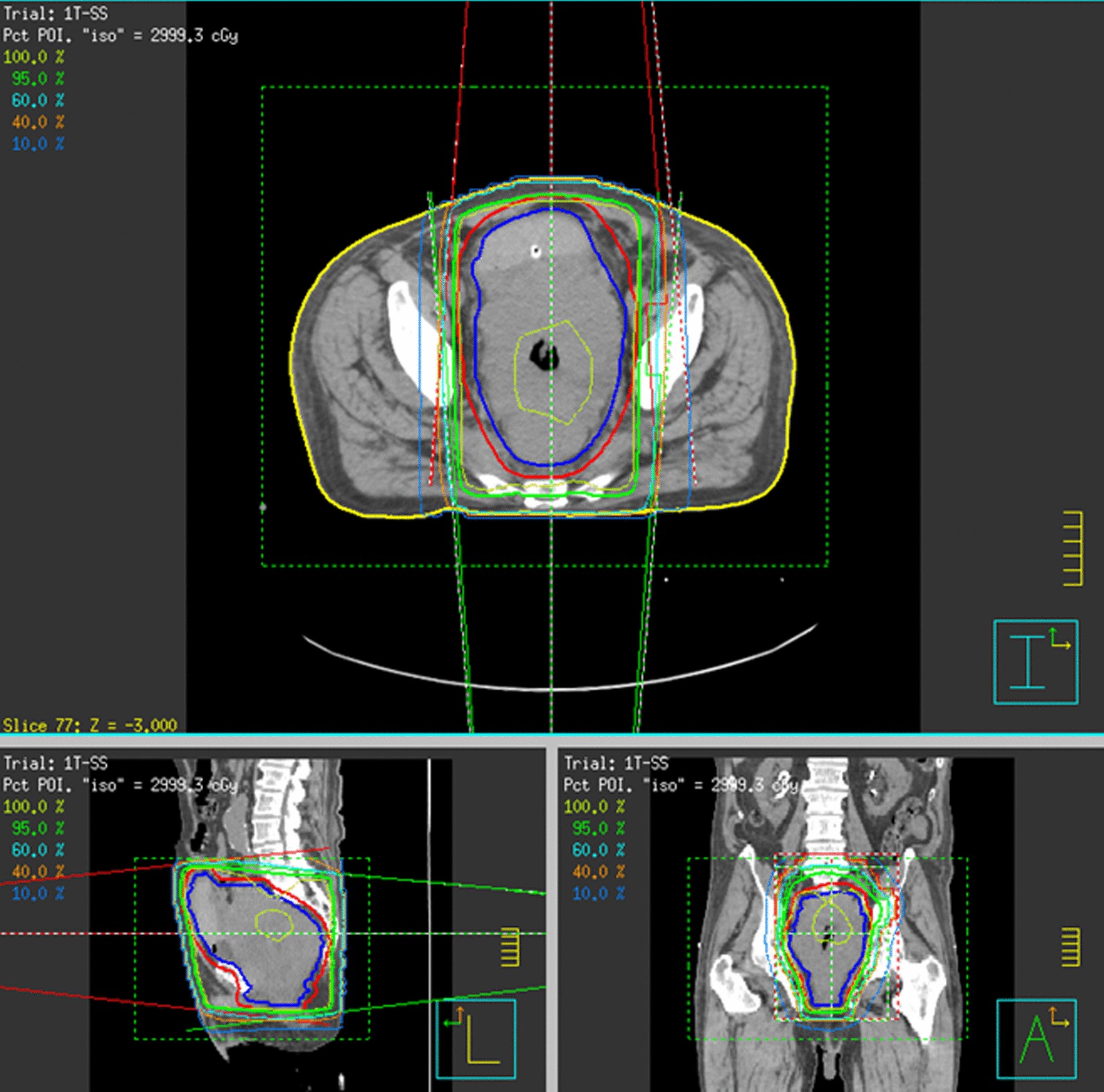
Radiotherapy 3D-CRT plan represented in three planes. A conventional anterior–posterior/posterior–anterior technique was used to target the pelvic tumor mass

In October 2017, a CT scan revealed substantially stable disease (SD) (Fig. 1b). During chemotherapy the patient was hospitalized several times for Grade 3–4 anemia due to repeated episodes of haematuria and rectal bleeding requiring repeated blood red cell transfusions. In September 2017 the patient was referred to our Radiation Oncology Unit for palliative pelvic RT due to persistent anemia. For RT, a standard palliative regimen of 30 Gy in ten fractions (3 Gy per fraction) using 18 MV photon beams in a conventional anterior-posterior/posterior-anterior technique (CT-based 3D-treatment plan) was prescribed. RT was delivered from 21/09/2017 to 04/10/2017 (Fig. 2).

In December 2017 a restaging total body CT scan revealed a very impressive response to RT: the pelvic mass volume was reduced from 850 cc to 105 cc, then without evidence of bladder and rectal infiltration (Fig. 1c).

As of March 2018, PSA decreased to 0.35 µg/L and in April 2018 the right nephrostomy was removed.

In July 2018, another total body CT scan showed further reduction of all tumor diameters (Fig. 1d). No locoregional or distant metastases were found.

In September 2018 the last nephrostomy was removed as well as the urinary catheter in January 2019. The patient was then followed with regular PSA value blood sampling and the last reported value was 0.13 µg/L (January 2020).

The patient is still on androgen deprivation therapy (ADT) with LH-RH analogues.

**Fig 3:**
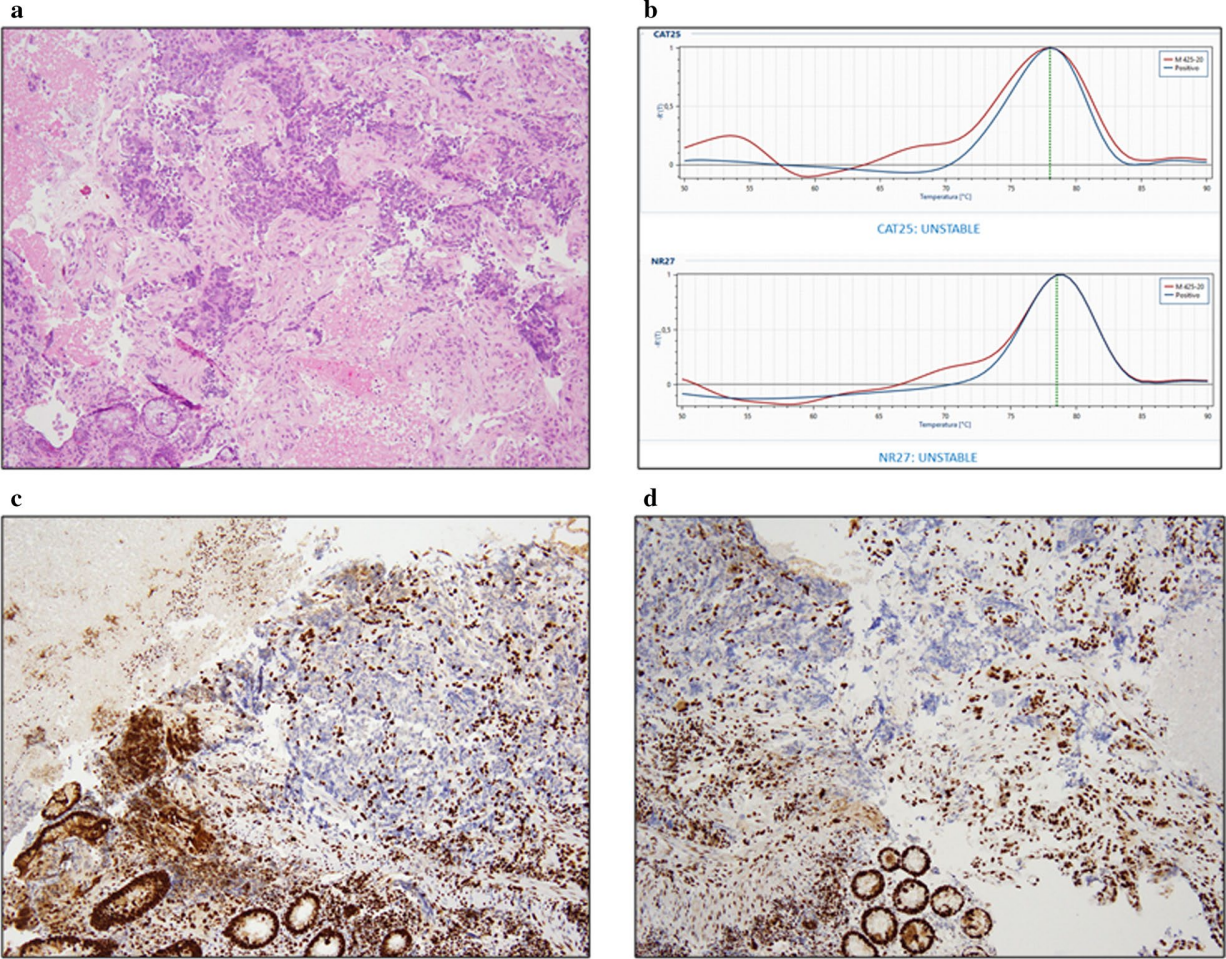
Tumor histology, microsatellite instability molecular analyses and IHC. **a** hematoxylin and eosin stain, **b** instability of CAT25 and NR27 markers, **c** immunohistochemistry for the MMR protein MSH 2, **d** immunohistochemistry for the MMR protein MSH 6. For the last two, background stroma and lymphocytes provide internal positive control

Molecular real time PCR analyses of biopsy showed high microsatellite instability and Immunohistochemistry showed an altered expression of MSH2 and MSH6 (Fig. 3a–d).

## Discussion

This represents, to our knowledge, the first reported case of an unusually dramatic and durable tumor response to palliative intent RT after SD obtained initially with 6 cycles of Docetaxel and ADT in PC patients.

MMR defects are represented in all tumor histology subtype (mostly endometrial, colorectal and gastric), and it is estimated that dMMR cancers are approximately 2–4% of all those diagnosed [8, 9]. Loss of MMR activity, due to the lack of function of any of its functional elements, is associated with tumor development and MSI. At the genomic level, dMMR tumors accumulate large numbers of frameshifts (FS) and single-nucleotide variants (SNV), and are therefore characterized by high mutational burden [10].

The peculiar genomic landscape of MSI tumors uniquely contributes to the quality of the tumor neoantigen profiles. Neoantigens result from FS and SNV, therefore an increasing number of FS and SNVs increases the probability that the tumor forms immunogenic antigens which can induce an immune response. Tumors with a fewer number of mutations are less likely to produce immunogenic antigens and probably will be less responsive to immunotherapy and vice versa [11]. Likely as a consequence, when compared with their microsatellite stable (MSS) counterpart, MSI tumors typically have a higher density of Th1 and effector-memory T cells, more in situ proliferating T cells, upregulated expression of immune checkpoints inhibitors (ICIs), including programmed cell death-1 (PD-1) and programmed cell death ligand-1 (PD-L1), and higher infiltration by mutation-specific cytotoxic T cells [12].

Based on the assumption that MSI plays a key role in the genetic tumor development and ongoing genetic modifications in established tumors, a potential correlation between response to ICIs and dMMR has been investigated. Le *et al.* evaluated immunotherapy with pembrolizumab in patients affected by MSI cancer (both CRC and non CRC) and MSS CRC. Objective response rates (ORRs) for MSI CRC and MSI non CRC were 40% and 71%, respectively, compared with 0% for microsatellite stable CRC [13]. These data suggest that MSI tumors might be particularly responsive to ICIs. Subsequent retrospective analyses have been conducted to confirm previous results [14]. Pembrolizumab was finally approved in May 2017 by the US Food and Drug Administration for unresectable or metastatic MSI-H solid tumor treatment, if progressed after standard treatment and in the absence of other valid alternatives.

The role of MSI in response to anti-PD-1 ICIs in PC was largely unknown because only one patient with PC was included in the series by Le *et al.* [13]. More recent [15, 16] demonstrated that pembrolizumab leads to responses or stable disease in subsets of patients with MSI-H CRPC, including those whose tumor expressing PD-L1. Although molecular features that contribute to response are currently not completely known, it seems now clear that MSI leads to an enhanced and effective immune system response, if stimulated by ICIs.

The role of RT in stimulating immune response has been investigated for a long time. To date we know that radiation causes DNA DSBs and Deng *et al.* suggested that RT damage plays a key role in stimulating interferon (IFN) expression, perhaps most efficiently by moderately hypofractionated RT regimens [17]. Furthermore Dewan *et al.* demonstrated that RT with hypofractionated regimens can stimulate “abscopal responses” when combined with ICIs [18]. Other studies hypothesized also that, in some cases, RT may successfully immunize the patient against the cancer, converting the irradiated tissue into an in situ* vaccine* and endowing the host with a set of new and powerful tools to control systemic disease [19].

Clinical experiences regarding the response of MSI tumors to RT are mainly retrospective.

Main evidence obviously relates to CRC and several studies showed no significant correlation between MSI and a better response to RT [20], contrary to what could be expected from the in-vitro analyses mentioned above [6, 7]. Further investigations have been conducted in the context of MSI endometrial cancer. These retrospective analyses again did not highlight a correlation between MSI and radiosensitivity [21], but rather showed a significant association between MSI and poorer 10-year local disease-free survival, disease-free survival, and cancer-specific survival [22] , while on the other hand an excellent response and even abscopal effects were reported for the combination of RT and ICIs [23]. Therefore, the correlation between RT response and MSI has yet to be clearly understood, as controversial results between preclinical and clinical data have been reported and as there are inconclusive data regarding the effect of RT only vs. a combination of RT and IT.

Our experience, however, suggests that investigation of MSI-status in these patients may be worthwhile in patient-specific treatment choice with minimal toxicity, either as monomodality treatment (for example in the case of RT with moderate doses to large volumes encompassing all macroscopic disease) or—even more likely to result in local and systemic response—as a combination of RT and IT [23], which may result in a dramatic benefit for these patients. The concept of applying moderate RT doses to macroscopic disease in combination with newer sensitizing systemic agents is currently investigated in various settings, with first promising results, for example for the combination of palliative dose RT and Trabectedin in patients with metastatic soft tissue sarcoma, having been reported recently [24].

## Conclusion

Our report adds to the overall evidence suggesting that extraordinary radiosensitivity may be encountered at least in a subset of MSI-H cancers and to our knowledge it is the first description of this phenomenon in a patient with MSI-H APC. This report therefore raises the possibility that also in these selected patients, in addition to their excellent response to RT alone, there is potential for synergy between RT and ICIs, having both potentially also systemic immune-enhancing effects as observed in other cancers. Given the low prevalence of PC with MSI, further investigations are necessary to better understand the effect of RT alone and in combination with immunotherapy. The value of MSI markers as predictive biomarkers should be formally evaluated further. Nevertheless, this and other reports suggest that routine evaluation of MSI in patients with a similar clinical situation is worthwhile and may change treatment strategy from a purely palliative approach to a quasi-curative paradigm.

## Data Availability

Not applicable.

## Abbreviations

ADT: Androgen deprivation therapy
APC: Advanced prostate cancer
CRC: Colorectal cancers
CT: Computed tomography
DDR: DNA damage response
DSBs: Double strand breaks
dMMR: Deficient DNA mismatch repair
FS: Frameshifts
ICIs: Immune checkpoints inhibitors
IFN: Interferon
LAPC: Locally advanced prostate cancer
LH-RH: Luteinizing hormone-releasing hormone
MSI: Microsatellite instability
ORRs: Objective response rates
PC: Prostate cancer
PD-1: Programmed cell death-1
PD-L1: Programmed cell death ligand-1
RT: Radiotherapy
SD: Stable disease
SNV: Single-nucleotide variants
